# The Institutional Worker

**Published:** 1920-02-21

**Authors:** 


THE
### INSTITUTIONAL WORKER
Being a Special Supplement to "The Hospital"

### OUR BUREAU OF INFORMATION.

### Rules for Correspondents.

1. Every letter must be accompanied by the coupon to be cut from the back cover (inside page) of The Hospital, current issue, and must contain the name and address of the correspondent with pseudonym for publication if desired. Replies by post cannot be given save under exceptional circumstances at the Editor's discretion.

2. Letters from Approved Homes in reply to special needs published in the Bureau should state terms and full particulars, and be sent prepaid under cover to the Editor of the Bureau with name written across coupon for identification.

3. Proprietors of Homes which have not yet been entered on the List of Approved Homes, but have spare accommodation likely to suit special needs, are invited to write for an application form for registration. The fee for registration, which includes two announcements of the Home in the Bureau and other privileges, is 10s.

4. All communications to be addressed to the Editor of The Hospital, 28 Southampton Street, Strand. London, W.C. 2, and marked "Bureau of Information."

### INSTITUTION FACTS AND FIGURES.

### The Question Box.

### THE UTILISATION OF VOLUNTARY HELP.

The question was :

**How would you deal with offers of voluntary part-time help in connection with your hospital from professional men, clerks, &c.? Give suggestions how their assistance might be utilised.**

**"F. S." replies:**

There is no limit to the amount of work capable of being carried out successfully by voluntary workers in our institutions.

I suggest that the best way of dealing with this matter would be by the formation of a National Association of Voluntary Hospital Workers, with county branches and district centres. There must be thousands of persons in the country whose services were given during the war to similar objects, and who now are unoccupied on account of the various war associations having ceased their activities. These thousands have gained just the right experience to fit them for the manifold duties they could undertake in hospital life. A national organisation would undoubtedly bring into the scheme the right class of worker and also the necessary amount of discipline required to attain efficiency of service. It would be desirable for members to give a fixed number of hours to the duties allotted to them in order that the hospital authorities might have a definite time-table to work upon. A suitable badge for members could be provided at small cost.

The following are some of the more obvious duties which could be undertaken :

Nursing.—A great number of ex-V.A.D. nurses would be available for working in the wards and other departments, and their services would help in reducing the hours of the permanent staff.

Dispensing.—Suitably qualified persons could be advantageously employed in making up stock mixtures, etc., under the supervision of one of the hospital pharmacists. There are many ladies who gained a sufficient knowledge of dispensing during the war capable of undertaking these duties.

Clerical work.—There is plenty of scope in this direction for all classes of clerks. The addressing of envelopes in large numbers for meetings, appeals, etc., registering of patients, etc., could be readily undertaken, thus releasing the permanent clerks for the duties for which they are specially trained. Clerks with a knowledge of accountancy would be of great assistance at the present time, when balance sheets and other financial statements are being prepared. A lady with clerical experience could be a valuable aid to the matron in dealing with her correspondence.

Gardening.—The local Garden Allotment Holders' Association might undertake the provision and upkeep of a kitchen garden in the hospital grounds by small gifts in kind and time by each of its members.

Cooking.—Much help could be given in the lighter work in the kitchen. Cooking classes in connection with the technical schools might easily combine instruction with a little charity and present their confectionery productions to the hospitals.

Sewing.—Good work is already undertaken by the Ladies' Garments Committees and needlework guilds in this direction, but useful work could be given by ladies helping in the hospital sewing-room in repairing linen and making uniforms, etc.

Valuable assistance in the directions stated above could undoubtedly be rendered through an association formed on a national basis. Such an organisation would be worthy of Royal patronage.

### INFANT WELFARE AT OLYMPIA.

The Ideal Homes Exhibition at Olympia has much that will interest nurses. It must be confessed that the model nurseries furnished by royal ladies are disappointing. They are merely doll's-house rooms, and, though gay and pretty, contain little or nothing that can help towards improved care for the health and comfort of the child. Only one has even a low table and "Montessori" small chairs, and not one contains the low washstand which is so valuable to a child, or cupboards where it can reach the toys, or any useful fitment to hold nursery first-aid and other appliances.

The Marylebone Infant Welfare Centre exhibits—very courageously—a set of small rooms in which work actually goes on during certain hours, real mothers bringing real babies to be weighed, and listening to real lectures, while one wall of the room is formed by a slowly moving procession of the general public. Many of the labour-saving contrivances will interest the nurse even more than others, and as to the model houses, she will ask, as all women ought to ask, "How if people are ill here?" Light, air, space, sufficient fresh water, and conveniences of various kinds need to be looked for beyond the questions of porches and concrete versus brick, and the interesting new ideas of elmwood walls and fireproof thatch.

### Massage Training.

If you will write to the Secretary of the Incorporated Society of Trained Masseuses, 157 Great Portland Street, London, W., she will send you a list of training-schools and private teachers, also any other information you require regarding fees for training, etc. We cannot give postal replies. A coupon should be enclosed with each inquiry.—D. M. C.

### Question for February.

### A NURSE'S VENTURE AS OPTICIAN.

**A trained nurse with long experience in an eye hospital, who has taken the certificate of the British Optical Association, is desirous of setting up business in a flourishing provincial town. Give suggestions and advice as to size of shop, its fittings, capital required, staff required, and the best method of making the shop known by advertising or otherwise.**

**A minimum payment of 10s. 6d. will be made for each published answer.)**

RULES.

The following rules must be observed:—

1. Contributions must be written on one side of the paper. Conciseness and terseness are desirable features. The MS. must bear the name and address of the sender and be accompanied by coupon to be cut from the back cover (inside page) of the current issue of The Hospital. A pseudonym must be chosen if the name is not to be published.

2. Contributions must be addressed to the Editor of The Hospital Institutional Worker Supplement, 28 & 29 Southampton Street, Strand, London, W.C. 2, should reach him before the end of the current month, and be marked in the left-hand corner "Facts and Figures."

### QUESTIONS INVITED.

In connection with our Question Box, we offer every month five shillings each for the best questions which are sent in for consideration. The questions may be on any institutional subject and concern any department, from the secretary's to the porter's, or the matron's to the domestic staff; the one essential is that they must be practical and deal with points that have a definite relation to hospital or institutional administration, upkeep, finance, or management. Points relating to artificers' work, housekeeping, the laundry, out-patients, and other practical matters will be welcome. It is hoped that thus, when difficult or doubtful points arise in the course of their work, institutional workers will be encouraged to put them in the form of a question and send them to the Editor, The Hospital Bureau of Information, 28 & 29 Southampton Street, Strand, London, W.C. 2, marked "Question Box."

The best questions will be published in due course, and our readers will be given the opportunity of answering them. Thus all institutional workers may be able to co-operate with the view of helping the work and smoothing the difficulties of each other. In short, we want the Question Box of the *Institutional Worker* to be freely used by every worker habitually.

### ENQUIRIES AND ANSWERS.

### Children and Infectious Illness.

### Accommodation Wanted for Paying Patients.

Mrs. E. M. J. Ward, the foundress of the Norland Institute, writes from 10 Pembridge Square, London, W. 2:—

I should be so glad to hear through your readers whether they know of any hospital or nursing home where we could send an infectious case from our Norland Nurseries, where we have twenty children between one month and eight years old. The children are particularly precious, their parents being abroad, and they are often only children of wealthy people. Under Government regulations we are not allowed to nurse these precious children in our own isolation rooms. We have for years been regular subscribers to the London Fever Hospital, but I am informed that, should this hospital be full, the child would be sent to the Metropolitan Asylums Board. Could not some qualified nurses start a hospital where such an institution as mine could pay a retaining fee and have the exclusive use of one room where one of our two matrons could go with the precious infant, and where private cases from wealthy homes could also be nursed?

### *EMPLOYMENT AND TRAINING.*

#### Nursing Shell-shock Patients.

Your best course will be to apply to the Matron-in-Chief, Pensions Service, 14 Great Smith Street, London, S.W. 1. There are several shell-shock hospitals under the Ministry of Pensions.—Nurse Mary.

#### Lectures on Tuberculosis.

We certainly recommend you to attend the course of lectures on Tuberculosis which will be given by the Hospital for Consumption, Brompton, starting on February 17 and every Tuesday and Friday afterwards during February, March, and April at 8 p.m. Apart from the lectures, practical instruction will be given in the laboratory and in the wards. Opportunities will be given also to see the dispensary practice in the out-patient department and to visit dispensary patients under the charge of the dispensary sister. The fee for the lectures alone is £1 1s., and £2 2s. for the complete course. An examination, which will be optional, will be held, and a certificate given at the end of the course. For any further particulars apply to the Matron of the Hospital.—R. N.

#### Army and Pensions Massage Association.

It has become necessary to disband the Army and Pensions Massage Association, as the scheme for associated action between the War Office and the Ministry of Pensions in the matter of the supply of massage personnel has been proved to be unworkable as far as the Ministry of Pensions is concerned. However, it will not affect you, inasmuch as those of its members who are in the War Office will continue to work as before, under the condition laid down in A.C. 1 of 1919 and its various amendments.—Sylvia.

#### I.S.T.M. Electrical Association.

You are too late to begin studying for the March examination in medical electricity which will be held by the Incorporated Society of Trained Masseuses early in March. The next examination will be in June next. The training is for three months.—I.S.T.M.

### *THE SICK AND IN NEED.*

#### Residential Accommodation for Nurse.

As you are a member of the College of Nursing, why not apply for accommodation to the Lady Superintendent of the Brighton and Hove Centre of the College of Nursing, Ltd., 45 Marine Parade, Brighton Residential Club? Enclose a stamped envelope for reply.—D. H.

#### Address Wanted.

The address you ask for is: The Women's Section of the League of Nations Union, 22 Buckingham Gate, London, S.W. The minimum subscription is 1s. Address your communication to the Secretary.—Loyal.

#### Pension Fund for Masseuses.

The Royal National Pension Fund for Nurses is open to masseuses holding recognised certificates. You can assure pensions of any amount and commence at any age. Premiums can be paid monthly or otherwise as is most convenient, so long as they are paid in advance. Write for full information to the Secretary, 15 Buckingham Street, Strand, London, W.C. 2.—Masseuse.

### *MISCELLANEOUS.*

#### Fire Lighters.

The very high cost of wood, and the difficulty until recently experienced in obtaining supplies, has called attention to substitutes for paper and wood for lighting fires. Experience shows, too, that but a small percentage of maids know how to build a fire correctly—thus much paper and wood is used to no purpose. A well constructed firelighter overcomes any difficulty in this direction. It is as cheap, if not a little cheaper, and, what is of greater importance, it saves time. Messrs. H. Freeman & Co., of 99 High Road, Tottenham, N. 15, supply a very reliable lighter. Though the price is somewhat higher than other makes, both material and construction are better than any other type we have tried. For this reason it is probably the most economical.—T. T.

